# Does origin of article impact citation metrics in *Gynecologic Oncology?*

**DOI:** 10.1016/j.gore.2022.100958

**Published:** 2022-03-11

**Authors:** Logan Corey, Christopher Walker, Alex Corey, Jonathan Konel, Ali Khalil, Hyejeong Jang, Seongho Kim, Radhika Gogoi

**Affiliations:** a Wayne State University School of Medicine, Detroit, MI, United States; b Karmanos Cancer Institute, Detroit, MI, United States; c University of Texas Southwestern Medical Center, Dallas, TX, United States; d RedHat, Inc, Raleigh, NC, United States; e St. Georges University School of Medicine, West Indies, Grenada

**Keywords:** Peer review, Double blind review, International contributors

## Abstract

**Objectives:**

To determine if the origin of article published in Gynecologic Oncology (“Journal”) is correlated with quality of the article when measured per US institution and per country, using an index of citation (IOC) metric as a stand-in for article quality.

**Methods:**

PubMed was used to query the Journal from 2005 to 2020. Articles not deemed original research were excluded. A US-only cohort (“US-Only”) was evaluated separately from the entire cohort (“Whole”). The IOC for each article was calculated by dividing the number of citations listed in PubMed by the days from the publication date to 9/1/2021. The IOC per US institution was summarized by the median value. All articles were hand reviewed for correctness. The Whole cohort included all countries with 3 or more publications (including all of the US-Only cohort) and underwent similar analysis. Correlation coefficients were estimated using Pearson’s correlation after log-transformation.

**Results:**

In the US-only cohort, 2733 articles from 276 institutions within the US contributed original articles to the Journal. The association between the number of publications per institution and the median IOC was not well correlated (Pearson’s Correlation coeffeicient r = 0.16, p = 0.009). In the Whole cohort, 5,848 original research articles were published from 40 countries. There was no difference between median IOC for articles from US compared to non-US institutions was (0.0026 vs 0.0027, p = 0.287). The US median IOC was ranked 17/40. The US accounted for just over half (51.2%) of publications, and there was a trend of decreasing Non-US publications over time (p = 0.0004).

**Conclusions:**

The Journal was fairly consistent in the quality of articles published over the 15-year study period when using the IOC as a surrogate for quality, regardless of the article’s country or US institution of origin.

## Highlights

• The adjusted citation score does not correlate with the volume of publications from US institutions.
• Articles from higher producing countries have higher adjusted citation scores.
• There is a decreasing trend in proportion of Non-US country contribution to *Gynecologic Oncology*.

## 1 Introduction

Peer review is a cornerstone of the research and publishing process that produces nearly a million manuscripts per year across the globe (Björk et al., n.d.). Unfortunately, there are data that the peer review process shows favoritism to well-known authors or institutions and from papers originating from the United States (Tomkins et al., 2017, Link, 1998)_._ Some medical journals in the field of Obstetrics and Gynecology and its subspecialties have implemented a Double Blind Review (DBR) process to blind the research team and the editor team from one another in an effort to mitigate these biases (Casway and Rouse, 2021, Journal of Gynecological Oncology, n.d.).

The gynecologic oncology subspecialty flagship journal, *Gynecologic Oncology* (henceforth referred to as “Journal”), has not adopted DBR (Elsevier. n.d.). Although a recent study showed that author gender biases are not apparent in the Journal, no studies have evaluated bias towards certain institutions or countries, and its corresponding impact on article quality (Penn et al., 2019). The implicit understanding of publication bias is that lower quality studies may be selected for publication for reasons other than the quality of the article’s contents.

The quality of an individual study or journal is not a definitive science and can be measured in a number of ways. The Journal has stated it uses Impact Factor (IF) as an indicator of success (Karlan, 2014). Impact Factor is calculated using the citations generated by a journal’s published articles and has been shown to be a reasonable estimator of medical journal quality (Saha et al., 2003). Our goal was to determine if the number of articles published per institution and country in the Journal correlated with quality of the articles, using an index of citation (IOC), defined below, as a stand-in for article quality.

## 2 Methods

### 2.1 Data gathering

PubMed was queried for ‘“Gynecol Oncol” [Journ]’ from 1/1/2005 to 08/01/2021 and a list of every article’s PubMed ID (PMID) was extracted. A web scraping program was built using Python to individually search each PMID within the PubMed database and abstract the articles’ first authors name, institution affiliation, country of origin, date of online publication, article type, and citation number. Only information from an author’s byline for that specific article was recorded. Only articles published online between 1/1/2005 and 12/31/2020 were included. Two cohorts were built and analyzed. The first cohort (“US-Only”) consisted of articles from institutions based in the United States (US). Every article from this cohort was manually reviewed by four of the authors (LC, CW, JK, AK) to ensure correct author byline information as well as article type and institutional affiliation. In most cases, the first author only had a single institutional affiliation. In cases where more than one affiliation was listed, Universities and Colleges took precedence. For example, if “Karmanos Cancer Institute” and “Wayne State University” were both listed in an author’s byline, the article was grouped under “Wayne State University.” In cases of uncertainty when the four reviewing authors could not agree, the senior author (RG) assigned the institution or the article was excluded from analysis. In instances when no institutions were listed, the article was excluded.

**Fig. 1:**
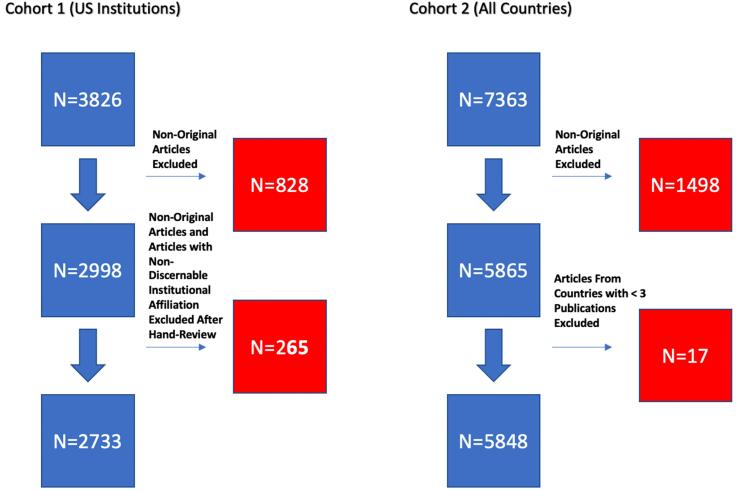
Flowsheet depicting exclusion of ineligible articles.

After the first cohort was built and audited to ensure 100% accurately abstracted information, a second cohort (“Whole”) was constructed that included all US and non-US countries with 3 or more publications with articles grouped according to the first author’s country listed in the byline. Countries with 3 or more publications were chosen to ensure an accurate median IOC was used, thus avoiding overweighting or underweighting of IOC score by countries with outliers either with an extremely high IOC or extremely low IOC. We only included articles with original research in both cohorts. Case Reports, Commentary, Editorials, Practice Guidelines, Published Erratum, Reviews and Society-sponsored statements were excluded. Meta-analyses were included. A PubMed citation score for each article was recorded on 9/1/2021. This study was deemed to involve non-human participants and was exempt from full review by the Wayne State University IRB. This process is outlined in Fig. 1.

### 2.2 Statistical methods

The index of citation (IOC) was calculated by dividing the number of citations for each article by the days from the online publication date to the data collection cuff-off date (Sept. 1, 2021). The IOC per institution or per country was summarized by the median value. Correlation coefficients were estimated using Spearman’s correlation after log-transformation.

## 3 Results

### 3.1 US-only cohort

**Fig. 2:**
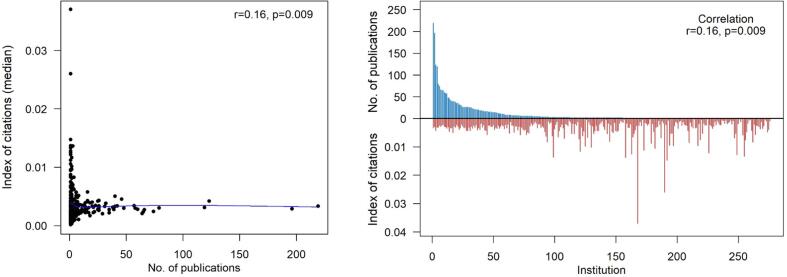
Association between number of publications per institution and median index of citation per institution. Spearman’s correlation was used to evaluate associations and reported as coefficients (r) and p-values (p). The blue solid line represents the loess curve fitted to the data. (For interpretation of the references to colour in this figure legend, the reader is referred to the web version of this article.)

**Fig. 3:**
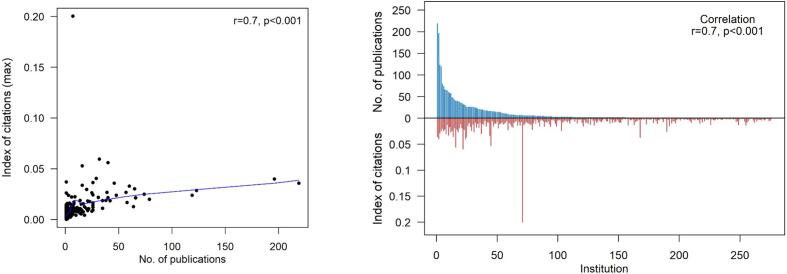
Association between number of publications per institution and maximum index of citation per institution. Spearman’s correlation was used to evaluate associations and reported as coefficients (r) and p-values (p). The blue solid line represents the loess curve fitted to the data. (For interpretation of the references to colour in this figure legend, the reader is referred to the web version of this article.)

After consolidation, there were 276 US-based institutions with an agreed-upon affiliation that contributed 2,733 original research articles to the Journal (Table 1). The difference in this number and the number of US publications in the Whole cohort is due to articles excluded after extensive hand review (articles without a clear institution listed in their online format). The median number of publications per US institution over the 15-year study period was 2 (1–219). The association between the number of publications per institution and the median IOC was not well correlated (r = 0.16, p = 0.009; Fig. 2). However, the association between the number of publications per institution and the maximum IOC of an article from that institution was very positively correlated (r = 0.7, p < 0.001; Fig. 3).

**Table 1:** Total number of publications and corresponding IOC stratified by US-institutiosn and all countries.

**US-Based Institutions**	
Number of institution – no.	276
Number of publications – no.	2733
Number of publications per institution – median (range)	2 (1,219)
Index of citation – median (range)	0.0030 (0.0002,0.2002)
Median index of citation per institution – median (range)	0.0028 (0.0002,0.0370)
Max index of citation per institution – median (range)	0.0092 (0.0002,0.2002)

**Countries with 3 or more Publications**	
Number of countries – no.	40
Number of publications – no.	5848
Number of publications per country – median (range)	42.5 (3,2998)
Index of citation – median (range)	0.0026 (0,0.2002)
Median index of citation per country – median (range)	0.0025 (0.0002,0.0036)
Max index of citation per country – median (range)	0.0159 (0.0027,0.2002)

### 3.2 Whole cohort (all countries)

There were 5,848 articles published from 40 countries (Table 1). The median number of publications per country was 42.5 (3-2998). The median IOC per country was 0.0025 (0, 0.0036). The US had the most original research articles (2998) followed by China (291), Canada (266), Italy (265), and Japan (241). Belgium had the highest median IOC of all countries (0.0035) with 3 or more publications. The US published just over half of the articles during the entire study period (51.2%) and had a median IOC that ranked 17th out of the 40 countries with 3 or more publications (Table 2).

**Table 2:** Summary of Publications by Country.

Country	No of Publications	Median IOC (Min, Max)
USA	2998	0.0026 (0.0000–0.2002)
China	291	0.0032 (0.0000–0.0488)
Canada	266	0.0031 (0.0000–0.0511)
Italy	265	0.0029 (0.0000–0.0354)
Japan	241	0.0025 (0.0000–0.0357)
South Korea	226	0.0030 (0.0000–0.0353)
Netherlands	175	0.0028 (0.0000–0.0231)
Germany	164	0.0027 (0.0000–0.0259)
France	145	0.0017 (0.0000–0.0508)
United Kingdom	128	0.0024 (0.0000–0.0268)
Australia	104	0.0032 (0.0000–0.0174)
Taiwan	83	0.0027 (0.0000–0.0142)
Denmark	71	0.0030 (0.0000–0.0189)
Spain	70	0.0019 (0.0000–0.0155)
Norway	63	0.0032 (0.0000–0.0119)
Austria	56	0.0019 (0.0000–0.0130)
Brazil	56	0.0019 (0.0000–0.0138)
Sweden	48	0.0027 (0.0000–0.0181)
Finland	46	0.0033 (0.0000–0.0110)
Greece	44	0.0015 (0.0000–0.0126)
Belgium	41	0.0035 (0.0000–0.0171)
Israel	41	0.0016 (0.0000–0.0131)
Turkey	36	0.0025 (0.0000–0.0104)
Poland	28	0.0026 (0.0000–0.0213)
India	27	0.0019 (0.0000–0.0185)
Czech Republic	26	0.0029 (0.0000–0.0179)
Switzerland	21	0.0023 (0.0000–0.0163)
Thailand	19	0.0025 (0.0000–0.0117)
Mexico	14	0.0023 (0.0003–0.0101)
New Zealand	8	0.0021 (0.0000–0.0087)
Singapore	8	0.0016 (0.0000–0.0087)
Chile	7	0.0036 (0.0000–0.0213)
Colombia	6	0.0028 (0.0008–0.0092)
Hungary	6	0.0016 (0.0000–0.0169)
Croatia	4	0.0015 (0.0000–0.0036)
Portugal	4	0.0021 (0.0020–0.0027)
Argentina	3	0.0020 (0.0020–0.0036)
Iran	3	0.0008 (0.0007–0.0039)
Slovenia	3	0.0006 (0.0004–0.0067)
South Africa	3	0.0002 (0.0000–0.0037)

The trend of Non-US publications decreased over the course of the study (Cochran-Armitage trend test p value = 0.0004, Table 3).

**Table 3:** Number of Non-US and US articles published by year. Cochran-Armitage trend (Non-US decreasing) test p value = 0.0006. The number of non-US publications tend to be decreasing over time (a trend test p < 0.001).

Publication Year	Non-US	US	Total
2005	316 (56%)	247 (44%)	563
2006	201 (50%)	198 (50%)	399
2007	195 (53%)	172 (47%)	367
2008	151 (45%)	186 (55%)	337
2009	147 (45%)	182 (55%)	329
2010	153 (52%)	139 (48%)	292
2011	212 (49%)	219 (51%)	431
2012	182 (49%)	187 (51%)	369
2013	192 (51%)	188 (49%)	380
2014	160 (46%)	185 (54%)	345
2015	157 (48%)	169 (52%)	326
2016	137 (42%)	188 (58%)	325
2017	144 (44%)	180 (56%)	324
2018	136 (46%)	159 (54%)	295
2019	145 (44%)	183 (56%)	328
2020	222 (51%)	216 (49%)	438

**Total**	**2850**	**2998**	**5848**

**Fig. 4:**
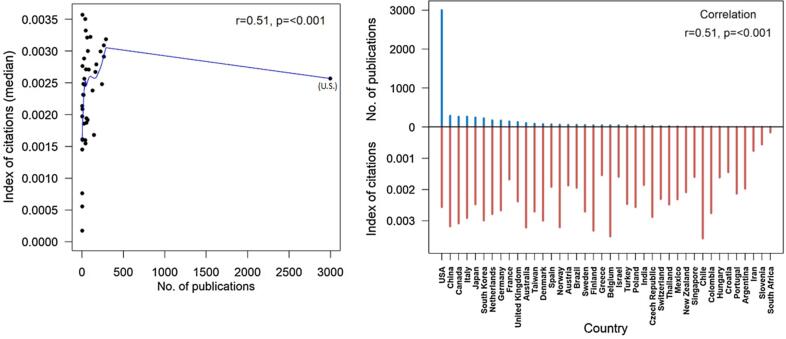
Association between number of publications per country and median index of citation per country. Spearman’s correlation was used to evaluate associations and reported as coefficients (r) and p-values (p). The blue solid line represents the loess curve fitted to the data. (For interpretation of the references to colour in this figure legend, the reader is referred to the web version of this article.)

When comparing all countries in the Whole cohort, the number of publications produced per country was associated with a higher median IOC (r = 0.51, p < 0.001; Fig. 4). This correlation persisted when comparing just non-US countries (r = 0.51, p < 0.001, S1). Lastly, meta-analysis was the type of article cited the most (data not shown).

## 4 Discussion

Our study found that most US institutions that contributed articles to the Journal do not do so regularly and there is a large range of productivity among institutions. Interestingly, the volume of publications did not correlate well with the median IOC score. That is, institutions that published infrequently in the Journal still produced articles that generated similar citation metrics compared to the articles from institutions of higher productivity. These results should be interpreted cautiously, but it does give credence that the Journal does a balanced editorial job of accepting articles from the US based on their content rather than their institution of origin. We did find that the more an institution publishes, the more likely the institution will produce an article that yields an unusually high IOC. The causes of this are not able to be identified with this study, but it may simply be a mixture of the idiom that “practice makes perfect” combined with laws of probabilities.

In the Whole cohort, we found that the number of articles produced by a country corresponded moderately well with the median IOC. Articles published from US institutions may be more favored as the US’ median IOC was middle of the pack (17/40) while accounting for over half of all articles contributed. Furthermore, since 2005, there has been a decreasing proportion of non-US countries contributing articles to the Journal. A better evaluation of a true trend in decreasing non-US articles may be the proportion of articles submitted versus articles published however these values are not publicly available.

The strengths of our study are that all original research articles over the last 15 years available online from the Journal were examined with nearly 6000 articles initially included. Additionally, these data measure the most important measurable real-world outcome: number of published articles and their corresponding citation metrics. The major limitation in our study is the inherent difficulty in measuring bias and measuring true research article quality. Using a standardized citation score is a reasonable metric for article quality, but is imperfect and may be misleading given post-publication promotion of certain articles over others, increased interest in some areas of research over others, or the ability of PubMed to have accurate citation numbers (Karlan, 2014).

This is the first study that has examined the Journal for institutional and national trends and corresponding citations generated per article. These results suggest the editorial process is simultaneously equitable to institutions within the US while also possibly containing some preference for US-based articles when compared to articles from non-US institutions. Our method of evaluation may be a useful tool for tracking trends in impact of articles at the individual country or institutional level. There are multiple barriers to implementing double blind review (hence why many organizations still do not offer this, including the Journal). The work of reviewers is already volunteer-based and the double-blind peer review process can increase administrative work and make submissions and reviews even more cumbersome and time consuming. Furthermore, in very specialized fields like gynecologic oncology, anonymity cannot always be guaranteed despite best efforts (Bazi, 2020). Additionally, DBR has drawbacks from an editorial standpoint including limiting ability to reading the prior work of the authors, evaluating material published by the submitter’s peers, and to assess for self-plagiarism and duplicative publishing. We agree that all attempts should be made to mitigate unconscious bias within practical reason.

In conclusion, the Journal was fairly consistent in the quality of articles published over the 15-year study period when using the IOC as a surrogate for quality, regardless of country or US institution of origin.

### CRediT authorship contribution statement

**Logan Corey:** Conceptualization, Data curation, Investigation, Methodology, Project administration, Resources, Supervision, Validation, Visualization, Writing – original draft, Writing – review & editing. **Christopher Walker:** Data curation, Investigation, Methodology, Project administration, Writing – review & editing. **Alex Corey:** Data curation. **Jonathan Konel:** Data curation, Writing – review & editing. **Ali Khalil:** Data curation. **Hyejeong Jang:** Formal analysis. **Seongho Kim:** Formal analysis, Writing – review & editing. **Radhika Gogoi:** Investigation, Methodology, Project administration, Supervision, Writing – review & editing.

## Declaration of Competing Interest

The authors declare that they have no known competing financial interests or personal relationships that could have appeared to influence the work reported in this paper.
