# Supplementary material for: Does origin of article impact citation metrics in Gynecologic Oncology?
Source: Gynecol Oncol Rep. 2022 Mar 11;40:100958. doi: 10.1016/j.gore.2022.100958 (PMC8933665; doi:10.1016/j.gore.2022.100958)
Supplement: Supplementary data 1 [file mmc1.pptx]

## Slide 1
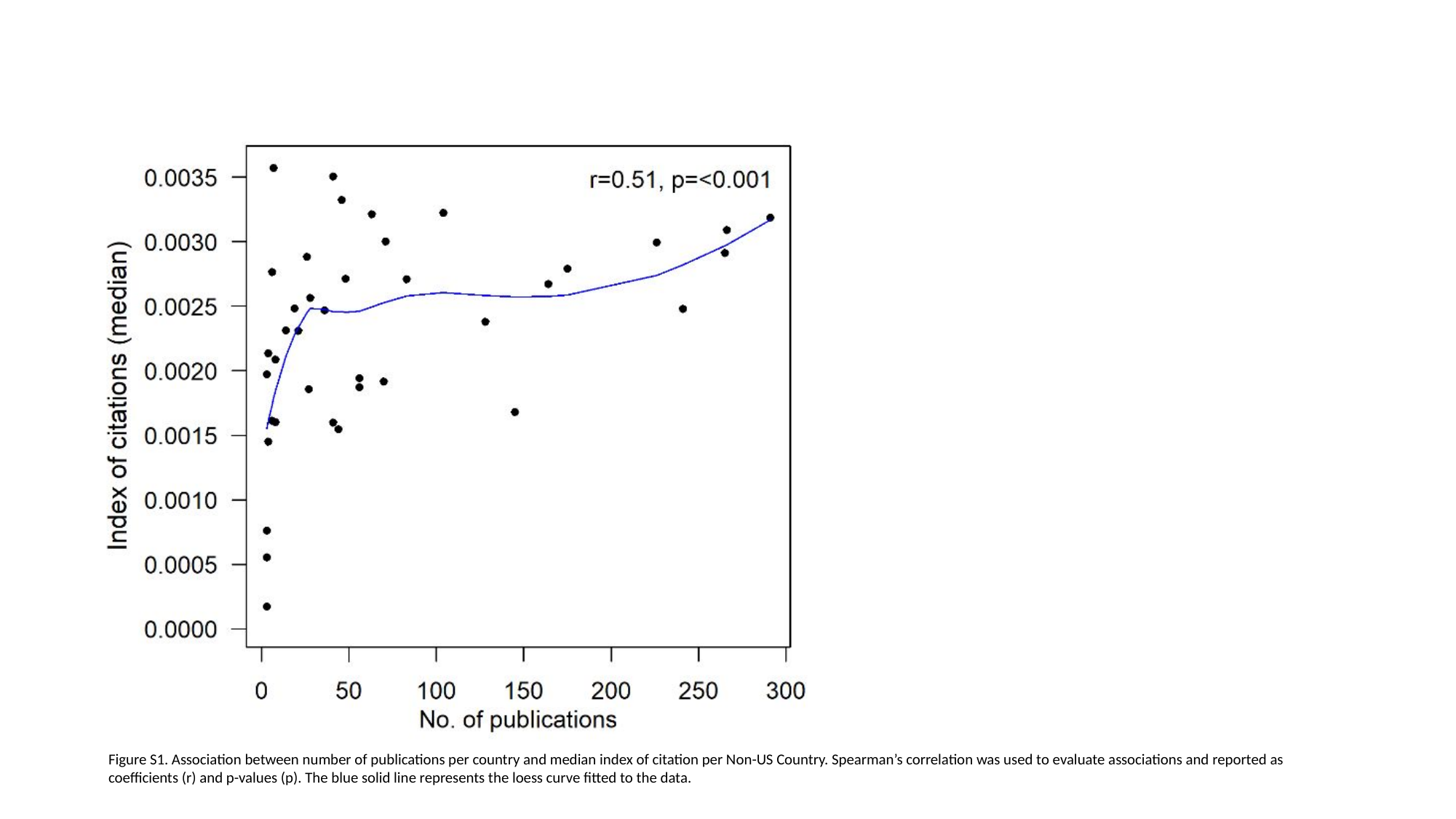

Figure S1. Association between number of publications per country and median index of citation per Non-US Country. Spearman’s correlation was used to evaluate associations and reported as coefficients (r) and p-values (p). The blue solid line represents the loess curve fitted to the data.
