# Supplementary material for: Does origin of article impact citation metrics in Gynecologic Oncology?
Source: Gynecol Oncol Rep. 2022 Mar 11;40:100958. doi: 10.1016/j.gore.2022.100958 (PMC8933665; doi:10.1016/j.gore.2022.100958)
Supplement: Supplementary data 2 [file mmc2.pptx]

## Slide 1
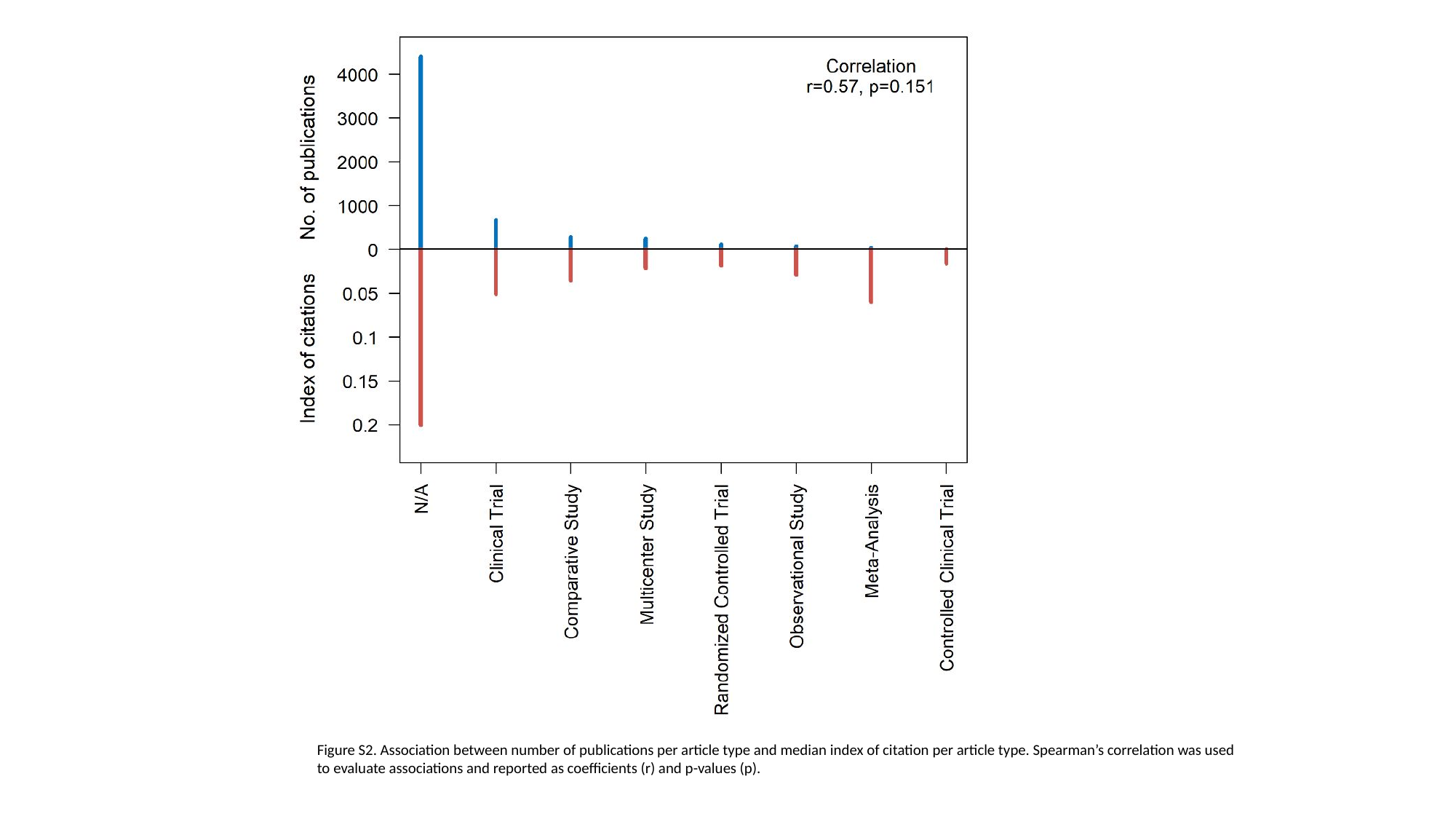

Figure S2. Association between number of publications per article type and median index of citation per article type. Spearman’s correlation was used to evaluate associations and reported as coefficients (r) and p-values (p).
